# Supplementary material for: Reconstructing the Temporal Origin and the Transmission Dynamics of the HIV Subtype B Epidemic in St. Petersburg, Russia
Source: Viruses. 2022 Dec 9;14(12):2748. doi: 10.3390/v14122748 (PMC9783597; doi:10.3390/v14122748)
Supplement: Supplementary file 1 [file viruses-14-02748-s001.zip › viruses-1866446-supplementary.pdf]

Genbank numbers:
OP441418,OP441419,OP441420,OP441421,OP441422,OP441423,OP441424,OP441425,OP441426,OP441427,OP441428,OP441429,OP441430,OP441431,OP441432,OP441433,OP441434,OP441435,OP441436,OP441437,OP441438,OP441439,OP441440,OP441441,OP441442,OP441443,OP441444,OP441445,OP441446,OP441447,OP441448,OP441449,OP441450,OP441451,OP441452,OP441453,OP441454,OP441455,OP441456,OP441457,OP441458,OP441459,OP441460,OP441461,OP441462,OP441463,OP441464,OP441465,OP441466,OP441467,OP441468,OP441469,OP441470,OP441471,OP441472,OP441473,OP441474,OP441475,OP441476,OP441477,OP441478,OP441479,OP441480,OP441481,OP441482,OP441483,OP441484,OP441485,OP441486,OP441487,OP441488,OP441489,OP441490,OP441491,OP441492,OP441493,OP441494,OP441495,OP441496,OP441497,OP441498,OP441499,OP441500,OP441501,OP441502,OP441503,OP441504,OP441505,OP441506,OP441507,OP441508,OP441509,OP441510,OP441511,OP441512,OP441513,OP441514,OP441515,OP441516,OP441517,OP441518,OP441519,OP441520,OP441521,OP441522,OP441523,OP441524,OP441525,OP441526,OP441527,OP441528,OP441529,OP441530,OP441531,OP441532,OP441533,OP441534,OP441535,OP441536,OP441537,OP441538,OP441539,OP441540,OP441541,OP441542,OP441543,OP441544,OP441545,OP441546,OP441547,OP441548,OP441549,OP441550,OP441551,OP441552,OP441553,OP441554,OP441555,OP441556,OP441557,OP441558,OP441559,OP441560,OP441561,OP441562,OP441563,OP441564,OP441565,OP441566,OP441567,OP441568,OP441569,OP441570,OP441571,OP441572,OP441573,OP441574,OP441575,OP441576,OP441577,OP441578,OP441579,OP441580,OP441581,OP441582,OP441583,OP441584,OP441585,OP441586,OP441587,OP441588,OP441589,OP441590,OP441591,OP441592,OP441593,OP441594,OP441595,OP441596,OP441597,OP441598,OP441599,OP441600,OP441601,OP441602,OP441603,OP441604,OP441605,OP441606,OP441607,OP441608,OP441609,OP441610,OP441611,OP441612,OP441613,OP441614,OP441615,OP441616,OP441617,OP441618,OP441619,OP441620,OP441621,OP441622,OP441623,OP441624,OP441625,OP441626,OP441627,OP441628,OP441629,OP441630,OP441631,OP441632,OP441633,OP441634,OP441635,OP441636,OP441637,OP441638,OP441639,OP441640,OP441641,OP441642,OP441643,OP441644,OP441645,OP441646,OP441647,OP441648,OP441649,OP441650,OP441651,OP441652,OP441653,OP441654,OP441655,OP441656,OP441657,OP441658,OP441659,OP441660,OP441661,OP441662,OP441663,OP441664,
